# *Bacillus thuringiensis* and nucleopolyhedrovirus combination against *Spodoptera litura* alters the cigar tobacco leaves microbiome during air curing

**DOI:** 10.3389/fmicb.2026.1855531

**Published:** 2026-07-15

**Authors:** Alhadi Adam, Ramzan Khan, Binrong Lu, Tianran Lin, Haoran Liao, Lingfei Peng

**Affiliations:** 1 Biological Control Research Institute, Fujian Agriculture and Forestry University, Fuzhou, China; 2 State Key Laboratory of Agricultural and Forestry Biosecurity, Fuzhou, China; 3 China Fruit Fly Research and Control Center of FAO/IAEA, Fuzhou, China; 4 Longyan Branch of Fujian Tobacco Company, Longyan, China

**Keywords:** *Bacillus thuringiensis*, cigar tobacco, microbiomes, nucleopolyhedrovirus, *Spodoptera litura*

## Abstract

**Introduction:**

*Spodoptera litura* continues feeding on cigar tobacco leaves during air curing, causing substantial economic losses. This study evaluated the synergistic efficacy of a *Bacillus thuringiensis* (Bt) and nucleopolyhedrovirus (NPV) combination for controlling *Spodoptera litura* and investigated its effects on the bacterial and fungal microbiomes of cigar tobacco leaves during air curing.

**Methods:**

Laboratory bioassays were conducted to determine the optimal Bt:NPV ratio and evaluate the synergistic toxicity. The selected formulation was subsequently validated under field conditions. Cigar tobacco leaves treated with the optimal Bt–NPV mixture were air-cured for 23 days, after which bacterial (16s rRNA) and fungal (ITS) communities were characterized using Illumina NovaSeq high-throughput sequencing.

**Results:**

The Bt:NPV mixture at a 4:1 ratio exhibited the strongest synergistic activity against second-instar *Spodoptera litura*, with an LC50 of 0.64 ml L^−1^ and a co-toxicity coefficient of 153.8. Bt–NPV treatment significantly altered the leaf microbiomes by reducing microbial richness while increasing microbial diversity and evenness. Compared with the control, treated leaves showed increased relative abundances of *Pantoea*, *Pseudomonas*, and Eurotiales, together with reduced abundances of *Staphylococcus* and Cladosporiales. These findings demonstrate that Bt–NPV treatment effectively controlled *Spodoptera litura* while reshaping bacterial and fungal community composition during air curing.

**Discussion:**

The synergistic Bt–NPV formulation represents an effective and environmentally friendly strategy for controlling *Spodoptera litura* during air curing while promoting an ecological framework that may support leaf preservation. However, the direct effects of Bt–NPV-induced microbiome changes on cigar tobacco quality, aroma development, and chemical maturation were not evaluated and should be investigated through integrated chemical, metabolomic, and sensory analyses in future studies.

## 1 Introduction

Tobacco (*Nicotiana tabacum L*.) is a highly important commodity worldwide because it sustains millions of farmers and is a significant industry in most countries. The process of converting raw leaves into a commercial product is quite complicated, and a significant effect on sensory quality is observed since fresh leaves are not initially consumed because of a high concentration of nicotine and unpleasant green odors (Hu et al., 2022). Nonetheless, cigar tobacco production is a prolonged process of air-curing, unlike tobacco, which needs flue-curing and is immediately dried at elevated temperatures. Moreover, the biochemical transformations facilitate the breakdown of alkaloids and carbohydrates into metabolites that make the product less harsh and construct the aroma (Yang et al., 2024). Recent research indicates that those changes are highly influenced by phyllosphere microbiomes (Vorholt, 2012; Zhai et al., 2020). The phyllosphere represents a complex and transient habitat where microbial inhabitants must adapt to rapid fluctuations in nutrient availability and surface chemistry. These microbial successions are not merely passive bystanders but are active participants in the leaf’s metabolic decline, determining the rate at which chlorophyll and complex proteins are degraded during the early stages of curing.

Furthermore, low environmental temperatures and humidity are necessary for microbial activity and also explain why cigar tobacco leaves are highly susceptible to attacks by *S. litura*; the larvae damage the leaves during the curing process, resulting in economic losses. Moreover, the limited airflow and the dense cover of leaves in curing structures may result in microclimates that not only promote microbial curing but also increase the speed of *S. litura* population growth and dispersion, requiring a quick and effective response. The integration of diverse biological control agents is increasingly recognized as a vital strategy for overcoming the limitations of single-agent applications, such as slow kill rates or the development of behavioral resistance in lepidopteran pests. Although biologically-based agents *Bacillus thuringiensis* (Bt) and nucleopolyhedrovirus (NPV) have proven to be effective and ecologically friendly, their use has been considered in terms of effects on the delicate microbial ecosystem (Montesinos, 2003; Hu et al., 2022).

*Bacillus thuringiensis* produces Cry toxins, which disrupt the insect midgut epithelium (Rabbee et al., 2019), and nucleopolyhedrovirus acts as an obligate insect pathogen, causing systemic viral infection of larvae (Elshaghabee et al., 2017; Hidalgo Martinez et al., 2020). The physiological explanation of the synergistic effect of such agents is that the toxins that form the pores of Bt can interfere with the intestinal barriers of the larvae, thus reducing the viral entry threshold and potentially reducing the time of death caused by the infection of NPV. Additionally, current research shows that Bt spores can remain on the leaf surface and alter the bacterial and fungal community structures (Zhang et al., 2008). However, the ecological impact of NPV remains less understood (Fuxa, 2004). Although the efficacy of these biopesticides under field conditions has been studied in many studies, there is still a significant gap in knowledge about their non-target effect on the microorganisms that are important to the quality of tobacco that grows after harvest, especially in the high-density environment of a curing house.

As cigar tobacco curing is very reliant on the chemical changes that occur under the influence of microbiomes, any interference with other microbial populations that may be induced by pest management measures can eventually influence the quality of tobacco. Moreover, the change in microbial community dominance between potentially pathogenic or spoilage-related groups and those with favorable fermentative activity is a major marker of successful curing. A biocontrol approach that balances this community and yet reduces the pest activity would provide a two-fold solution to the cigar tobacco industry, where physical leaf integrity is accompanied by high-value chemical maturation (Li et al., 2024). More importantly, it is not known whether the combination of Bt–NPV is synergistic against tobacco cutworm and whether it is compatible with the curing process without any adverse impact on the microbial succession and chemical compositions.

To overcome these uncertainties, the study sought to determine the toxicity of the combination of Bt–NPV on *Spodoptera litura* and its effects on the dynamics of the bacterial and fungal community during the natural air curing of cigar tobacco leaves. Through bioassays and microbial analysis, the current research offers a complete evaluation of the compatibility between Bt–NPV-based pest management and cigar tobacco curing and quality formation.

## 2 Materials and methods

### 2.1 Insect rearing

*Spodoptera litura* pupae were purchased from Henan Jiyuan Baiyun Industrial Co., Ltd. and reared at the Biological Control Research Institute, Fujian Agriculture and Forestry University. Insects were maintained in an artificial climate chamber at 27 ± 1 °C, 75 ± 5% relative humidity, and a 14:10 h (L: D) photoperiod. After adult emergence, the moths were allowed to mate and oviposit freely.

The larvae’s first and second instars were fed organic sweet potato leaves, the third, fourth, fifth, and sixth instars were fed artificial diets, and the adults were provided with a honey solution (Gupta et al., 2005; Maharjan et al., 2023). A total of five generations were reared throughout the experiment for use in the bioassays.

### 2.2 Toxicity of *Bacillus thuringiensis* and nucleopolyhedrovirus against *Spodoptera litura* under laboratory conditions

The biopesticide, *Bacillus thuringiensis* var. *kurstaki*, was purchased from Shandong LuKang Biological Pesticide Co., Ltd., with a concentration of 8,000 IU mL^−1^, and nucleopolyhedrovirus was purchased from Guangzhou New Scenery Biological Engineering Co., Ltd., with a concentration of 10^9^ PIB mL^−1^.

Second instar *Spodoptera litura* larvae were used to evaluate the toxicity of Bt and NPV individually. Larvae were exposed to seven concentrations for each agent: 58.81, 19.61, 6.55, 2.18, 0.73, 0.245, and 0.079 mL L^−1^, prepared from the respective stock solutions, along with an untreated control. Based on LC_20_ values of Bt (0.13 mL L^−1^) and NPV (0.12 mL L^−1^), Bt–NPV mixtures were prepared on a toxic-unit basis at ratios of 1:1, 1:2, 2:1, 1:4, and 4:1 (Bt: NPV), while maintaining a constant total volume of 0.25 mL L^−1^ (sum of the LC_20_ values of Bt and NPV) (Maqsood et al., 2019).

The *Bacillus thuringiensis* and nucleopolyhedrovirus formulations were applied by spraying onto the surface of the artificial diet used for feeding *Spodoptera litura* larvae. The artificial diet was prepared by cooking a mixture of agar powder in water until it dissolved, then adding yeast powder as a nutrient, linseed oil as a lipid supplement, and vitamin C as an essential antioxidant. Sodium benzoate and soluble chlortetracycline hydrochloride were also added. The thoroughly mixed diet was allowed to cool and solidify before use (Gupta et al., 2005). Each concentration level and the control consisted of four replicates, with ten second-instar larvae allocated per replicate (40 larvae per concentration gradient). Consequently, a total of 320 larvae (40 larvae × 7 concentrations + 40 control larvae) were evaluated for each independent biopesticide assay.

### 2.3 Field experiment

The field experiment was conducted in Liancheng County, Longyan City, Fujian Province, China, for controlling *Spodoptera litura in cigar* tobacco by the application of a combination of Bt–NPV biopesticides. Based on prior laboratory bioassays that identified the Bt–NPV 4:1 ratio as having the highest synergistic mortality rate, this specific ratio was selected for field application.

For the field tests, an application rate of 0.64 mL L^−1^ (0.52 mL L^−1^ Bt and 0.12 mL L^−1^ NPV) served as the median concentration. To determine field toxicity across a broader spectrum, a total of five concentrations were prepared through adjusting this median rate by factors of 2 and 4 (specifically 0.25x, 0.5x, 1x, 2x, and 4x the median concentration) while maintaining the constant 4:1 mixing ratio. The evaluation of the optimal 4:1 Bt–NPV mixture comprised a gradient of five distinct mixed concentrations alongside an untreated control group. Each mixture concentration and control group was evaluated using four replicates, each containing ten second-instar larvae, for a total of 240 larvae used to map the combination toxicity curve.

To ensure uniform initial pest pressure and eliminate confounding variables from patchy wild populations, second-instar *Spodoptera litura* larvae were introduced onto the field-grown cigar tobacco via controlled artificial inoculation. The untreated control parts served as the natural baseline to observe larval behavior and feeding progression under ambient field conditions, and to calculate corrected mortality against natural background death. Although artificial inoculation may not fully represent the spatial heterogeneity and population dynamics of natural infestations, it is a standard field-entomology approach that enables controlled comparisons among treatments and improves the accuracy of efficacy assessments.

Mortality was recorded 4 days after the application. The LC_50_ values obtained under field conditions represent operational efficacy influenced by environmental factors rather than mechanistic toxicity parameters.

### 2.4 Curing house experiment

In June 2025, freshly harvested cigar tobacco leaves of the Haiyan 101 variety were collected from the field in Liancheng County, Longyan City, Fujian Province, China. To minimize individual leaf variation, each leaf was cut along the main vein into two parts (using the half-leaf method) (Zong et al., 2022). Then, the optimal concentration ratio of 4:1 (Bt: NPV), 0.64 mL L^−1^, was applied thoroughly to the harvested leaves, and other leaves were left as the control group.

The *Bacillus thuringiensis* and nucleopolyhedrovirus biopesticides-treated and untreated leaves were dried and cured in a natural air curing chamber for 23 days. During this period, the temperature and humidity were monitored, which were 25 °C to 35 °C, and 65 to 80%. After the curing was done, samples were collected for microbial and chemical analysis.

### 2.5 Microbial community analysis

#### 2.5.1 DNA extraction, PCR amplification, and high-throughput sequencing

The 16S rRNA and ITS1 sequencing were performed by Genesky Biotechnologies Inc. (Shanghai, China). A total of six samples were analyzed, which consisted of three biological replicates of the Bt–NPV-treated cigar tobacco leaves and three biological replicates of the control sample, which had their genomic DNA extracted using the FastDNA SPIN Kit.

An appropriate proportion of spike-in mixture with known gradient copy numbers was added to the sample DNA. The V5-V7 hypervariable regions of the 16S rRNA gene and spike-ins were amplified with the primers 799F (5′-AACMGGATTAGATACCCKG-3′) and 1193R (5′-ACGTCATCCCCACCTTCC-3′), and the ITS1 hypervariable regions of the fungal community and spike-ins were amplified with the primers ITS1 (5′-CTTGGTCATTTAGAGGAAGTAA-3′) and ITS2 (5′-GCTGCGTTCTTCATCGATGC-3′), and sequenced using an Illumina NovaSeq 6000 sequencer, and then the QIIME2 was applied to the raw read sequences (Bolyen et al., 2019; Callahan et al., 2016).

Cutadapt was used to trim the adaptor and primer sequences. Then, the quality control was performed using DADA2, and amplicon sequence variants were identified (Yang et al., 2018; Youssef et al., 2009).

The taxonomic assignments of ASV representative sequences were performed with a confidence threshold of 0.7 using a pre-trained Naive Bayes classifier trained on the SILVA (version 138.2) database. Subsequently, spike-in sequences were identified, and reads were quantified (Jiang et al., 2019).

### 2.6 Statistical analysis

Median lethal concentrations (LC_50_) and 95% confidence intervals of the concentration were estimated via probit analysis. Dose–response relationships were fitted by linear regression of probit-transformed mortality against log_10_-transformed concentrations, from which regression equations, slope values, and correlation coefficients were obtained using IBM SPSS Statistics for Windows, Version 26.0, and graphical outputs were prepared using the Python programming language. The dose–mortality relationship was described using the regression equation: Y = a + b log_10_ (C), in which Y is the probit-transformed mortality, a is the intercept, b is the slope, and C is the concentration of Bt or NPV.

The co-toxicity coefficient (CTC) technique was used to test the interaction of Bt and NPV. The calculation of CTC values was done following the method of Sun and Johnson (1960), who stated that CTC values above 120 denote synergism, CTC values between 80 and 120 denote additivity, and CTC values below 80 denote antagonism, and the equation is: $\mathrm{CTC}=\frac{\text{Expected}\;\mathrm{LC}50}{\text{Observed}\;\mathrm{LC}50}\times 100$ .

The relative effectiveness and toxicity of the treatments were estimated using the Effective Treatment Index (ETI). ETI was calculated by dividing the concentration of the active ingredient of the treatment that achieves the desired effect by the concentration of the control that achieves the same baseline effect, and the equation is: $\mathrm{ETI}=\frac{\text{Expected}\;\mathrm{LC}50}{\text{Observed}\;\mathrm{LC}50}\times 100$ .

All statistical analyses were performed using IBM SPSS Statistics for Windows, Version 26.0 (IBM Corp., Armonk, NY, United States) and Python visualization. A significance level of *p* < 0.05 was used throughout the study.

## 3 Results

### 3.1 The toxicity of Bt against *Spodoptera litura* larvae under lab conditions

The laboratory bioassays demonstrated that the larval mortality increased with the increase in the Bt concentration. The regression analysis revealed a strong dose–response relationship, with a coefficient of determination (*R*^2^ = 0.916) and regression equation *Y =* 0.04 + 0.81*X*. These results indicate that Bt is highly virulent to early larval stages. Probit regression analysis further confirmed a consistent dependent mortality response (Table 1).

**Table 1:** Joint-action analysis and toxicity parameters of single Bt, single NPV, and the optimized 4:1 Bt–NPV combination against second-instar *Spodoptera litura* larvae.

Biopesticides	LC_50_ (mL L^−1^)	95% confidence limit	Slope ± SE	Chi-square	ETI	CTC	Interaction
Bt	0.99	(0.85–1.16)	1.87 ± 0.22	1.94	–	–	–
NPV	0.96	(0.82–1.12)	1.79 ± 0.19	2.11	–	–	–
Bt–NPV (4:1)	0.64	(0.53–0.76)	2.41 ± 0.27	1.68	100.6	153.8	Synergistic

Full corrected mortality values for all five tested Bt: NPV mixing ratios are summarized in the Results section to verify the 4:1 ratio delivers maximum larval mortality and the strongest synergistic interaction.

### 3.2 The toxicity of NPV against *Spodoptera litura* larvae under lab conditions

A clear dose-mortality relationship was observed between nucleopolyhedrovirus and second-instar *S. litura* larvae. The regression analysis showed a strong fit with a coefficient of determination (*R*^2^ = 0.959), and the regression equation *Y* = 0.03 + 0.92*X* indicates a reliable dose–response relationship under laboratory conditions. The probit analysis confirmed a consistent concentration-dependent mortality response (Table 1).

### 3.3 The toxicity and synergistic effect of the Bt and NPV combination against *Spodoptera litura* larvae under lab conditions

The *Bacillus thuringiensis*, nucleopolyhedrovirus, and their mixture (Bt–NPV, 4:1 ratio) toxicity against *Spodoptera litura* larvae was evaluated using probit analysis and joint-action metrics (Table 1). The LC_50_ value of the (Bt: NPV, 4:1) mixture was lower (0.64 mL L^−1^) compared to individual treatment, indicating higher toxicity_._ The regression analysis showed a dose–response relationship with a correlation coefficient (*R*^2^ = 0.871) and regression equation *Y* = 1.09 + 1.98*X*.

Preliminary screening assays were conducted using LC_20_-based toxic-unit mixtures of Bt and NPV at ratios of 1:1, 1:2, 2:1, 1:4, and 4:1. The corrected mortality values varied among the tested combinations, with the Bt: NPV (4:1) mixture treatment producing the highest corrected mortality (90.53%), followed by 1:4 (87.37%), 1:1 (55.79%), 1:2 (55.79%), and 2:1 (45.26%). These results clearly demonstrated the high efficacy of the 4:1 mixture and provided the basis for selecting this ratio for subsequent probit toxicity and joint-action analyses.

The expected toxicity index (ETI) based on the proportional contribution of Bt and NPV in the mixture was 100.6, and the corresponding co-toxicity coefficient (CTC) value was 153.8, exceeding the threshold, which caused severe leaf damage. This sustained larval activity confirms that the open-field environment was fully hospitable to the *Spodoptera litura* life cycle, providing a clear baseline that highlights a threshold value of 120 and indicating a synergistic interaction.

Compared with individual treatments, the Bt–NPV mixture demonstrated enhanced toxicity against *S. litura* as evidenced by a lower LC_50_ value and a steeper regression slope. The combined application of Bt–NPV was therefore more effective than its individual application, suggesting a synergistic effect under laboratory conditions.

### 3.4 Toxicity of Bt and NPV combination against *Spodoptera litura* larvae in the field

In the untreated control parts, the inoculated larvae successfully established themselves on the cigar tobacco, continued their feeding cycles, and experienced the strong protective efficacy of the Bt–NPV treatment. Furthermore, the bioassays showed that the combination of Bt–NPV formulation (4:1) was effective against *S. litura* larvae under field conditions, in which larval mortality was increased with the increase in concentrations, indicating a clear dose–response relationship. Probit regression analysis estimated an LC_50_ value of 1.6 mL L^−1^ water for the Bt–NPV (4:1) treatment.

### 3.5 High-throughput sequencing analysis

A total of 199 bacterial ASVs were detected in Bt–NPV-treated amplicons and 401 bacterial ASVs in control amplicons. In the case of fungi, 74 ASVs were observed in Bt–NPV-treated samples and 44 ASVs in control samples.

#### 3.5.1 Alpha diversity analysis

**Figure 1:**
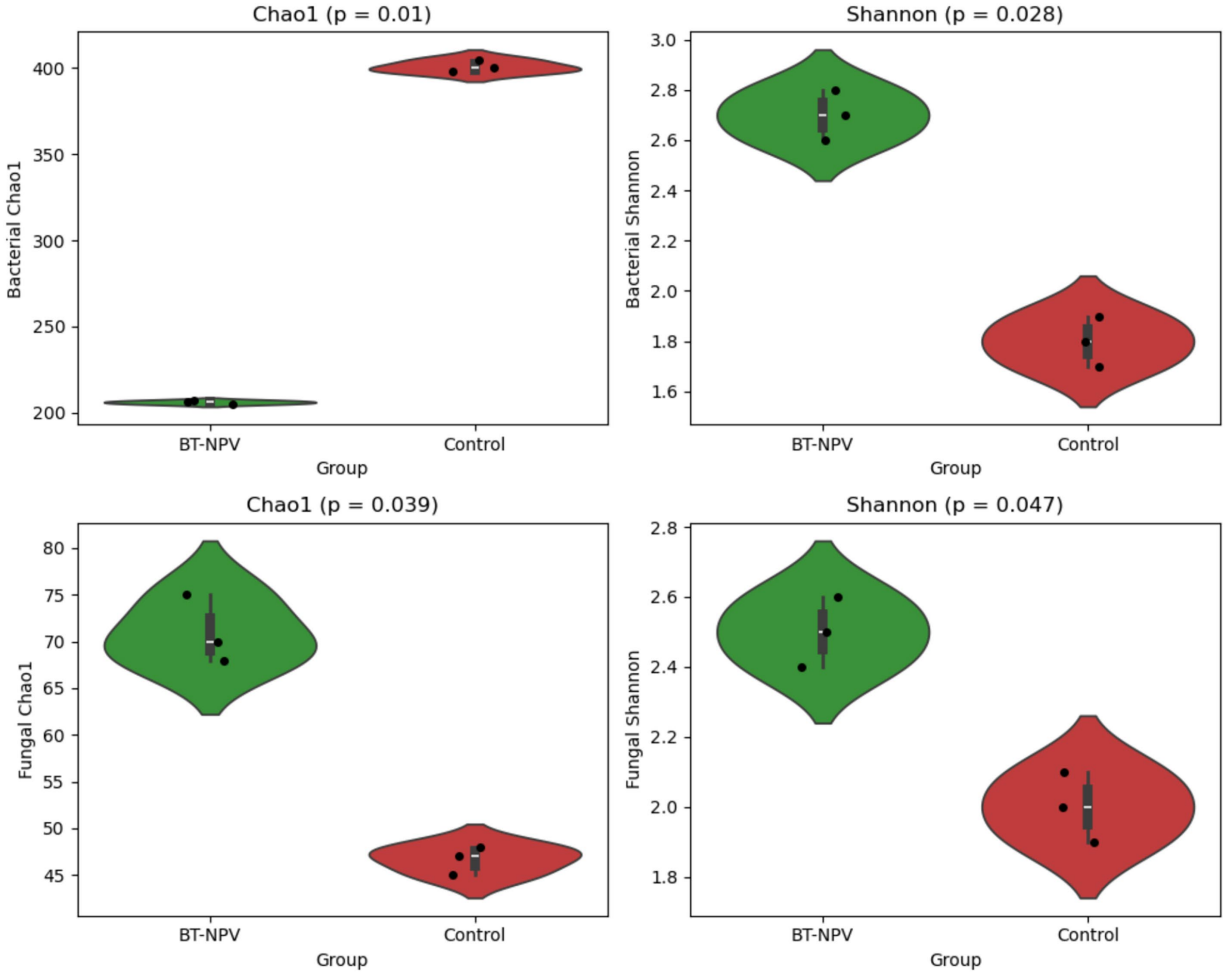
Comparison of bacterial and fungal alpha diversity metrics in Bt–NPV-treated and control cigar tobacco leaf samples.

The Wilcoxon rank-sum test was used to compare alpha-diversity indices between the Bt–NPV-treated and the control samples. Bacterial and fungal richness (Chao1) was significantly lower in Bt–NPV-treated samples compared to the control; these differences were statistically significant (Wilcoxon test: *p* < 0.01 for bacteria and *p* < 0.038 for fungi). However, diversity and evenness were significantly higher in the treated samples (Wilcoxon test: *p* < 0.028 for bacteria and *p* < 0.047 for the fungal community) (Figure 1).

#### 3.5.2 Beta diversity analysis

**Figure 2:**
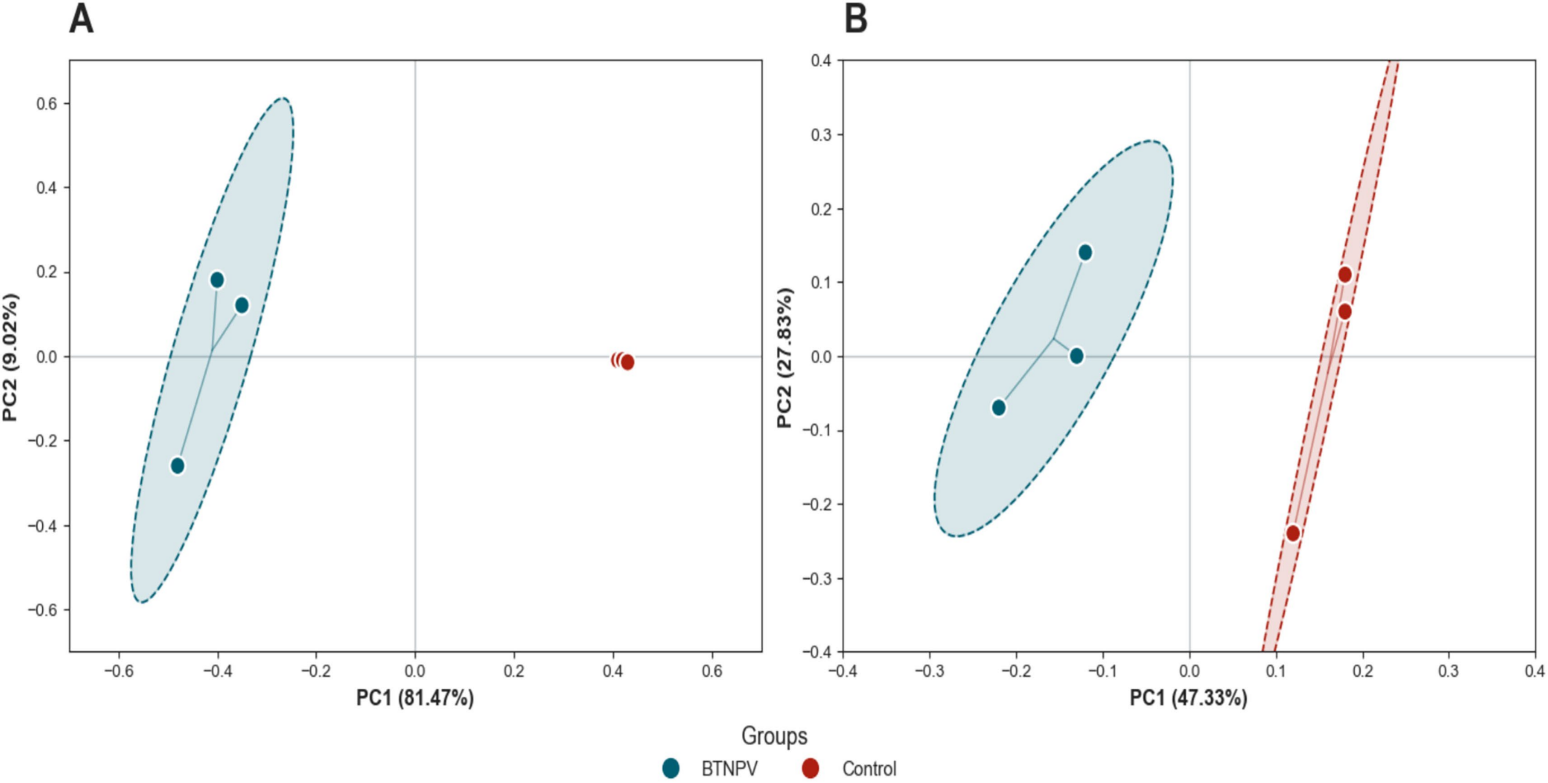
Beta diversity analysis. **(A)** PCoA analysis of bacterial community. **(B)** PCoA analysis of fungal community.

The beta diversity analysis was done using Bray-Curtis distances, which found that there was a distinct separation of Bt–NPV-treated and control samples. The principal component analysis (PCA) and principal coordinates analysis (PCoA) indicated different groupings of samples based on treatment in the bacterial and fungal communities (Figure 2).

Multivariate analysis of variance (PERMANOVA) showed that treatment was a significant means to account for the variation in community composition. In the case of bacterial communities, the treatment effect was significant (*R*^2^ = 0.42, *p* = 0.03). Equally, the composition of the fungi varied greatly among treatments (PERMANOVA, *R*^2^ = 0.38, *p* = 0.04).

**Figure 3:**
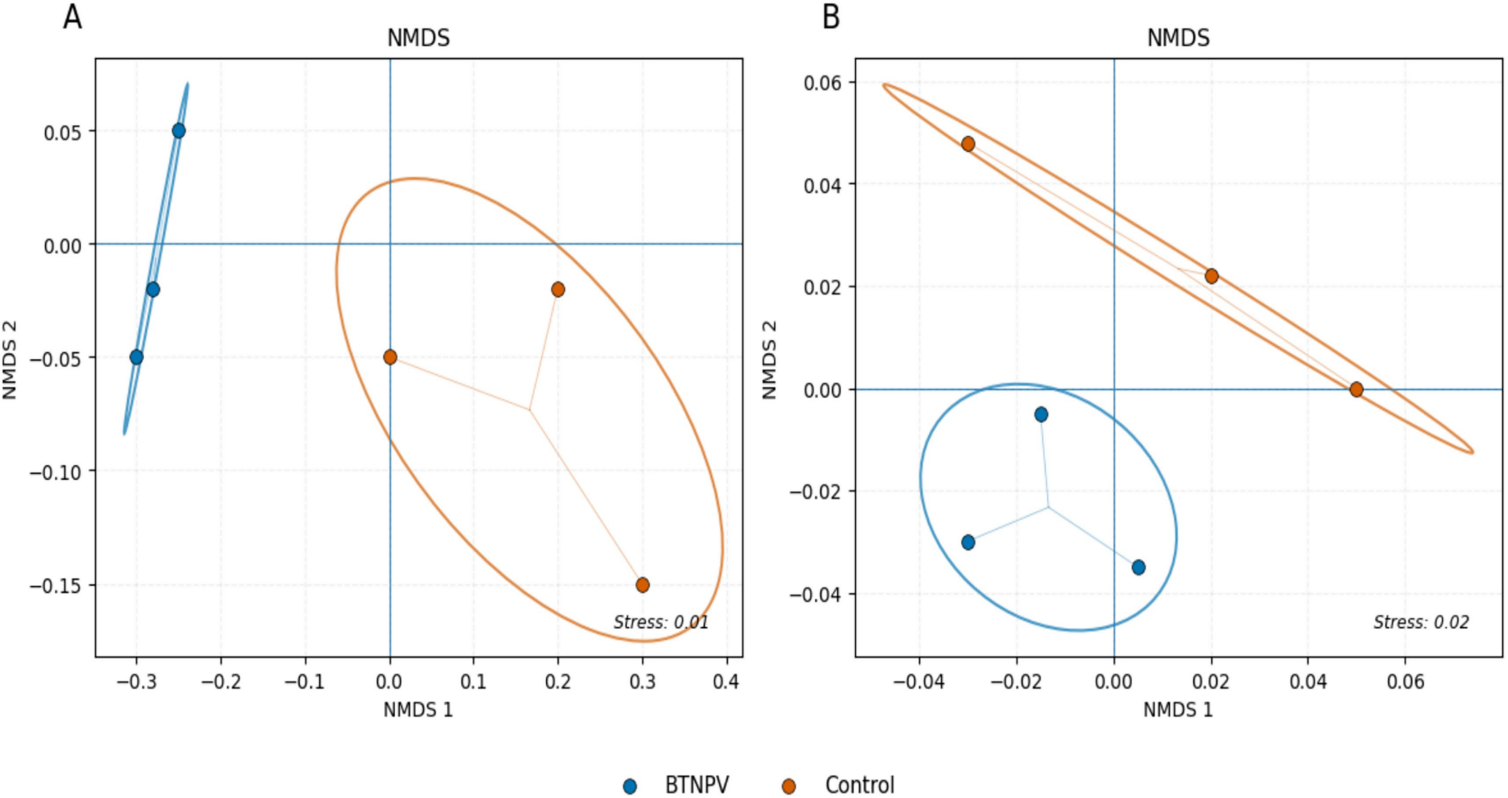
Non-metric Multidimensional Scaling (NMDS) ordination based on Bray-Curtis distances for **(A)** bacterial communities and **(B)** fungal communities on cigar tobacco leaves.

To further validate the shifts in microbial community structure, Non-metric Multidimensional Scaling (NMDS) was performed for both bacterial (Figure 3A) and fungal (Figure 3B). The bacterial NMDS plot revealed a distinct distinction of the treatment groups, whereby the Bt–NPV-treated samples moved along the NMDS1 axis as the samples moved away from the control groups as the curing continued. Likewise, the fungi community exhibited a high level of clustering based on treatment and time. The segregation of the treated and the control group was especially high during the air curing phase, and it can be concluded that the synergistic use of Bt and NPV played a major role in the assembly and successional progression of the fungi phyllosphere microbial community. The restructuring of the phyllosphere microbiomes was statistically robust, as evidenced by the Non-metric Multidimensional Scaling (NMDS) ordination, which achieved a high degree of fit (Stress = 0.02).

#### 3.5.3 The relative microbiome community after natural air curing

**Figure 4:**
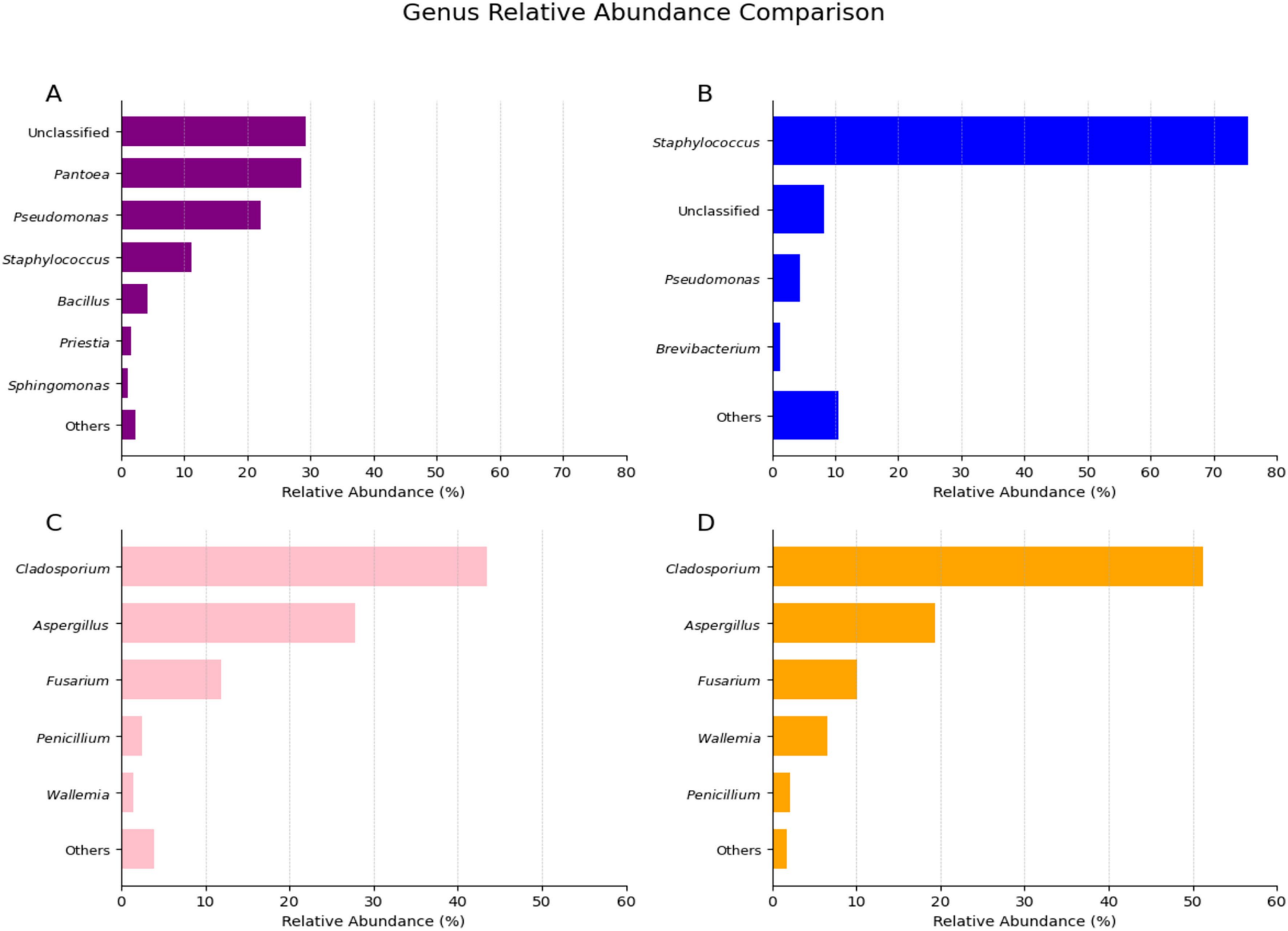
The relative abundance of bacteria and fungi. **(A)** Bacterial genus distribution in Bt–NPV samples; **(B)** Bacterial genus distribution in Control samples; **(C)** Fungal genus distribution in Bt–NPV samples; **(D)** Fungal genus distribution in Control samples.

As shown in (Figure 4A), the relative abundance of bacteria at the genus level in the Bt–NPV-treated cigar tobacco leaves is dominated by *Sphingomonas* (1.09%), unclassified reads (29.09%), *Staphylococcus* (11.29%), *Pantoea* (28.67%), *Priestia* (1.28%), *Pseudomonas* (22.35%), *Bacillus* (4.12%) and other (2.05%), In contrast, the control samples are dominated by *Staphylococcus* (75.54%), unclassified reads (8.27%), *Pseudomonas* (4.38%), *Brevibacterium* (1.05%), and others (10.59%) (Figure 4B), which shows that the bacterial community is more diverse in the Bt–NPV-treated sample compared with the control.

The relative abundance of fungi at the genus level in the Bt–NPV-treated cigar tobacco leaf is dominated by *Aspergillus* (27.95%), with minor contributions from *Penicillium* (2.34%), *Wallemia* (1.17%), *Fusarium* (12.04%), *Cladosporium* (43.46%), and others (3.79%) (Figure 4C). In contrast, the control sample is dominated by *Aspergillus* (19.37%) and *Cladosporium* (51.34%), followed by *Wallemia* (6.6%), *Fusarium* (10.05%), *Penicillium* (1.92%), and other genera (1.64%) (Figure 4D).

#### 3.5.4 The dynamic changes of the bacterial community

We analyzed the composition of the bacterial community of cigar tobacco leaves across various taxonomic ranks-phylum, class, order, and genus- to compare control samples and the cigar tobacco leaves treated by the Bt–NPV biopesticide.

**Figure 5:**
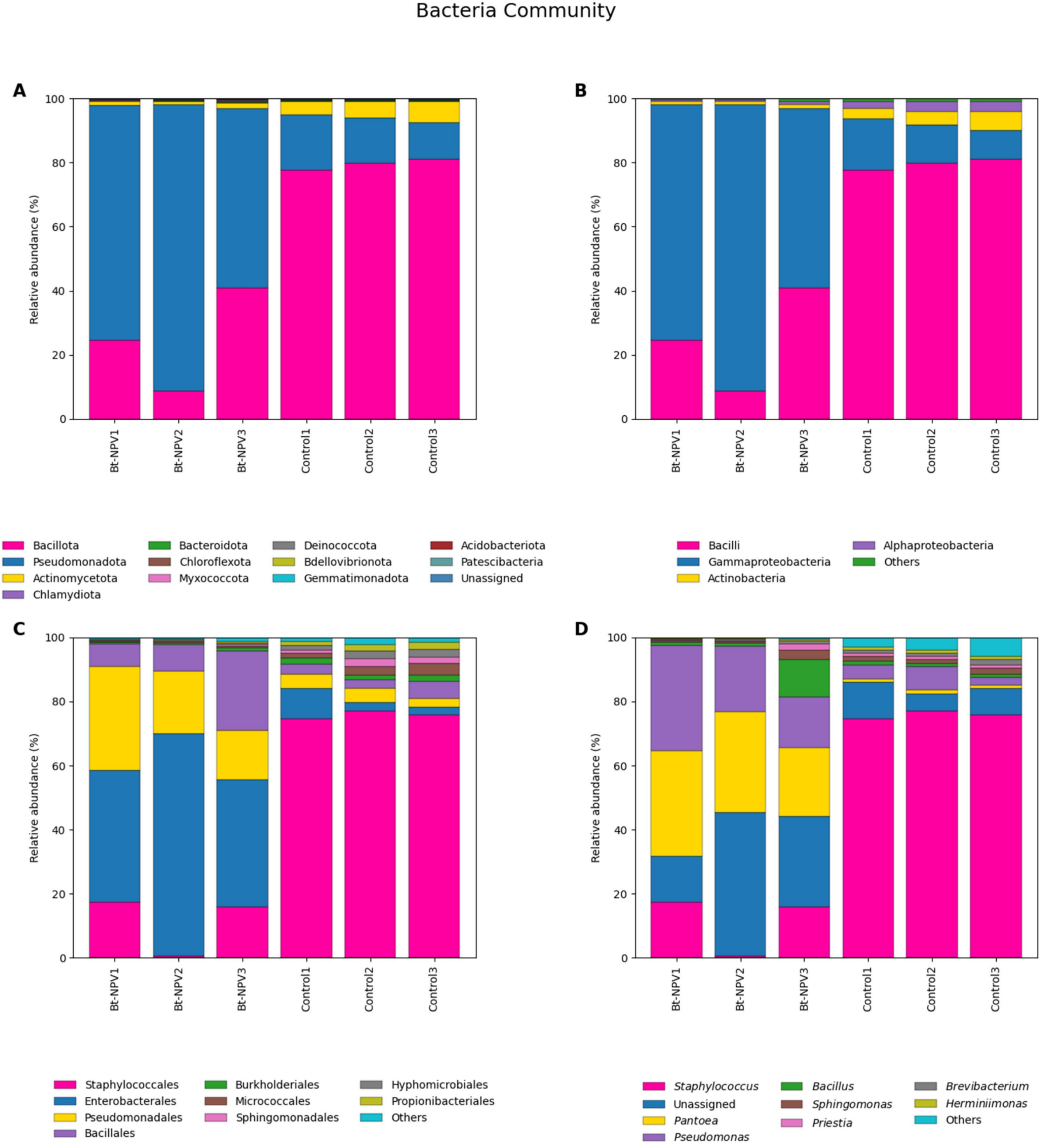
The relative abundance of bacterial communities at different levels: **(A)** phylum, **(B)** class, **(C)** order, and **(D)** genus.

As in (Figure 5A), at the phylum level, both groups contain the major taxa Bacillota and Pseudomonadota, with minor percentages of Actinomycota. However, there is a significant difference between their relative distributions. In the control group, the community is dominated by Bacillota (80%), while the relative abundance of Pseudomonadota is low (12%). However, in Bt–NPV-treated samples, there is a pronounced preference for Pseudomonadota (70%), with a significant decrease in Bacillota (25%).

At the class level, Bacilli are the largest proportion of the community (80%) in the control group, which is also in line with the prevalence of Bacillota. On the other hand, Gammaproteobacteria (72%) are significantly enriched in Bt–NPV-treated samples, whereas the Alphaproteobacteria and Actinobacteria are mostly minor contributors in both samples (Figure 5B).

On the order level, the differences between treatments are even more apparent. Control samples have high percentages of Staphylococcales and a slight percentage of other orders. The samples that have been treated with Bt–NPV have higher levels of Enterobacterales, Pseudomonadales, and Bacillales, however. This decrease of Staphylococcales in the treated leaves and the increase in the number of Enterobacterales, Bacillales, and Pseudomonadales indicate the shift of the community that is dominated by one taxon to a more diverse and balanced form. This long-term survival of Bacillales and, in others, even growth might be indicative of the robustness of sporogenic lineages of *Bacillus* in the presence of biopesticide-related stress (Figure 5C).

The control bacteria at the genus level are highly biased toward *Staphylococcus* (75.54%), with minimal role taken by *Pantoea*, *Pseudomonas, Bacillus, Sphingomonas*, and *Brevibacterium*. The samples that have undergone treatment with Bt–NPV, on the other hand, demonstrate a more uniform community, with a higher abundance of *Pantoea, Pseudomonas, Bacillus, Sphingomonas, Brevibacterium*, and a few unidentified genera (Figure 5D). Altogether, these structural changes are supported by microbial diversity patterns. There is very high dominance by one genus (*Staphylococcus*) in the control samples, which leaves evenness low. The application of Bt–NPV treatment diminishes this domination and allows increasing the number of different genera of Pseudomonadota, creating a more diverse community.

**Figure 6:**
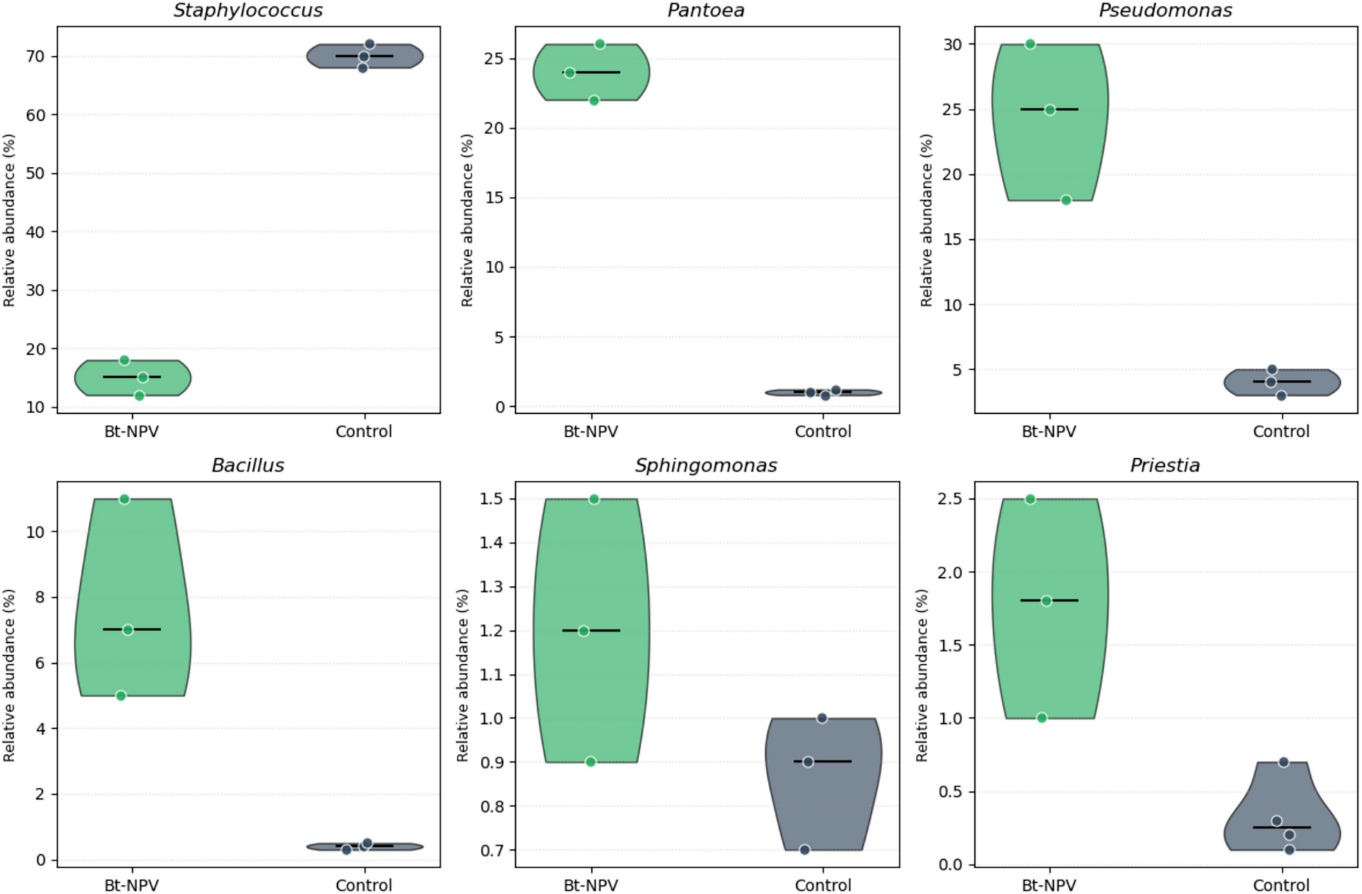
The relative abundance comparison of the dominant bacterial communities at the genus level.

These results demonstrate that the Bt–NPV treatment acts as a selective ecological filter, actively reshaping the cigar tobacco phyllosphere by suppressing dominant *Staphylococcus* taxa and facilitating the proliferation of beneficial, metabolically robust Pseudomonadota genera such as *Pantoea* and *Pseudomonas*. This diversification is probably due to the competitive release after it suppressed the dominant Bacillota taxa. Collectively, the Bt–NPV treatment was also connected with significant changes in the cigar tobacco bacterial community, such as a decrease in the relative abundance of *Staphylococcus* and an increase in the relative abundance of *Pantoea* and *Pseudomonas* (Figure 6).

#### 3.5.5 The dynamic changes of the fungi community

**Figure 7:**
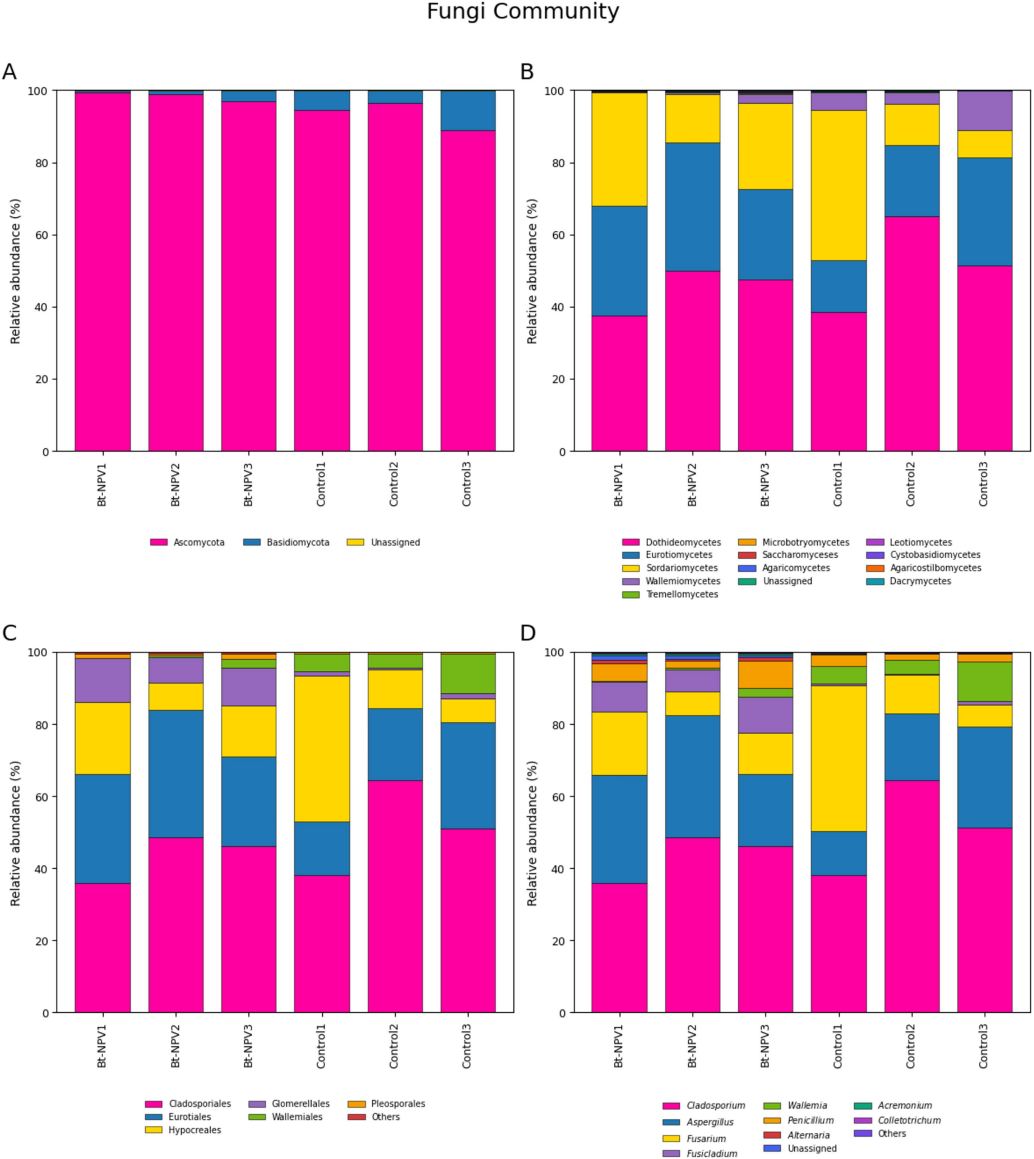
The relative abundance of fungi communities at different levels: **(A)** phylum, **(B)** class, **(C)** order, and **(D)** genus.

The comparative profile of the composition of the fungal community at four taxonomic levels, phylum, class, order, and genus, of the control and Bt–NPV-treated cigar tobacco leaves. At the phylum level, Ascomycota predominated in both groups highly, accounting for 94% of the fungal community in control and slightly higher, 98%, in the Bt–NPV-treated samples. Basidiomycota is following a similar pattern, with a drop of 5 to 1.5% in the control compared to the treated (Figure 7A).

At the class level, the prevailing classes are Dothideomycetes, Eurotiomycetes, and Sordariomycetes. Dothideomycetes decreased by 51% in the control to 45% with Bt–NPV treatment; Eurotiomycetes increased by 23 to 30%, and Sordariomycetes increased by 22 to 25%. The minor classes are not significantly different between groups (Figure 7B).

At the order level, greater alterations are seen at the order level. The dominant order Cladosporiales in the control (50%) decreased to 43% following Bt–NPV treatment. The change in Eurotiales is significant (23 to 32%), whereas it decreases in Hypocreales (20 to 14%). Additionally, Glomerellales increased (from 2 to 6%) and Wallemiales (from 7 to 3%) (Figure 7C).

At the genus level, the control community was characterized by *Cladosporium* (51.34%), *Aspergillus* (19.37%), and *Fusarium* (10.05%). *Cladosporium* reduces to 43.46, and *Aspergillus* grows significantly to 27.95 after Bt–NPV treatment. *Fusarium* is reduced to 12.04%, and *Fusicladium* is raised to 6%. *Wallemia* is reduced to 6.6% by 3%, and *Penicillium* is slightly raised (2.34) (Figure 7D).

**Figure 8:**
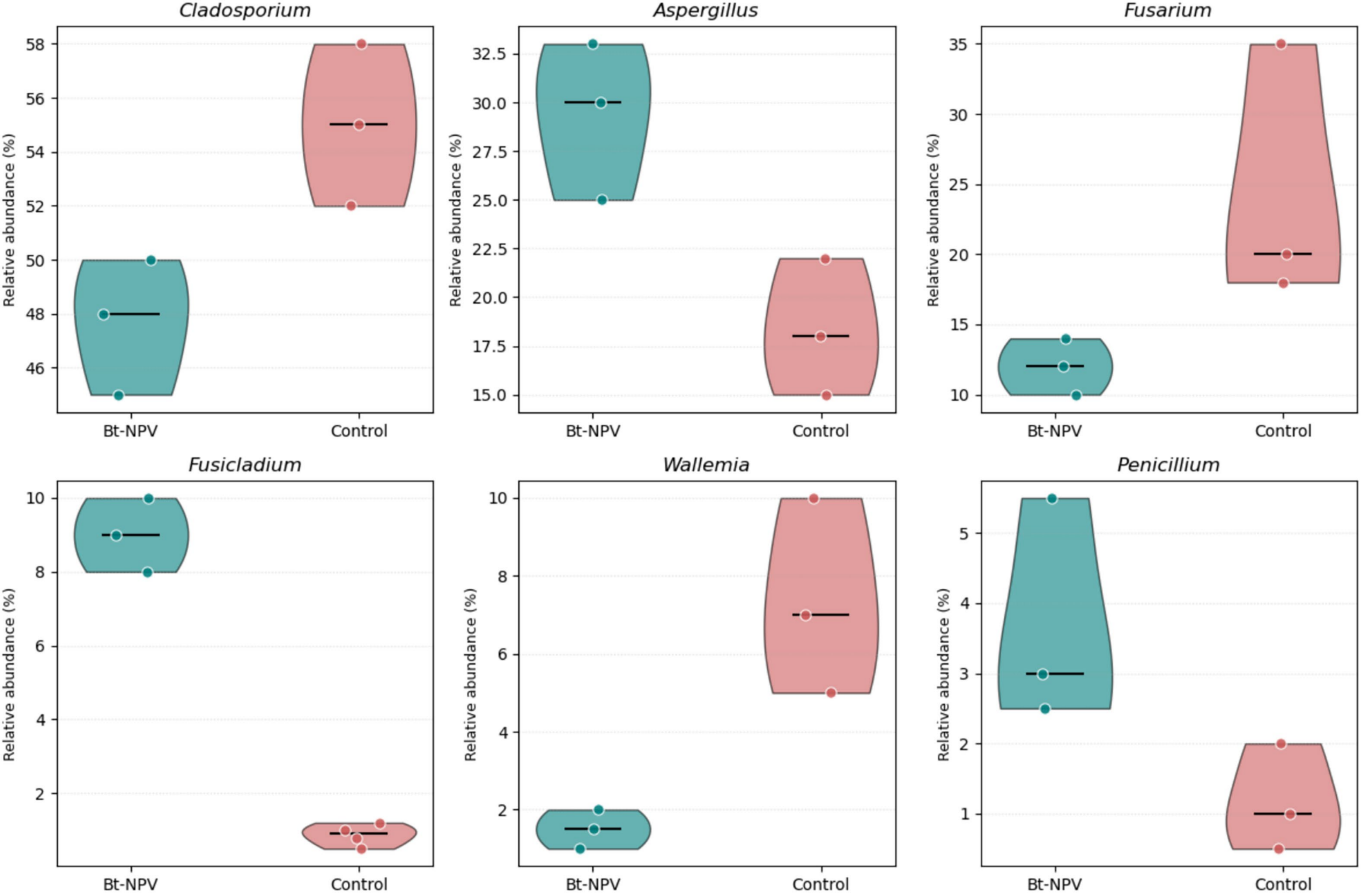
The relative abundance comparison of the dominant fungi communities at the genus level.

These results show that the treatment with Bt–NPV had an impact on the relative abundance of specific fungal taxa, which showed an increase in the proportion of *Aspergillus*, *Fusicladium*, and *Penicillium*, and a decrease in the proportion of *Cladosporium*, *Fusarium*, and *Wallemia* (Figure 8).

## 4 Discussion

This study demonstrates that a low concentration of *Bacillus thuringiensis* and nucleopolyhedrovirus combinations can be used to provide excellent control of *Spodoptera litura* and, at the same time, alter the microbial community. Furthermore, the observation period, a 4-day (96 h) period, is traditionally considered short for evaluating individual nucleopolyhedrovirus dynamics due to the virus’s characteristically slow replication and incubation period. However, the rapid mortality achieved within 96 h in this study highlights the physiological mechanism underlying the synergistic acceleration of the Bt–NPV combination, in which *Bacillus thuringiensis* endotoxins rapidly form lytic pores in the midgut epithelial cells of *Spodoptera litura* larvae, disrupting the peritrophic membrane barrier. This structural breach allows the NPV occlusion bodies or budded virions to bypass the primary gut defenses, significantly accelerating systemic viral infection and shortening the typical incubation period. Therefore, evaluating efficacy within a compressed 4-day period is critically relevant for post-harvest air curing; rapid suppression is essential to halt larval feeding before the cigar tobacco leaf moisture content drops, thereby directly preserving structural leaf integrity.

Moreover, the exogenous biological materials, Bt spores and NPV viral occlusion bodies, that induce this remodeling include shifts in the leaf surface microhabitat. Our findings regarding the microbiome community shifts build upon existing literature, for instance, Wang et al. (2014) found that Bt sprays had no impact on total microbial biomass of leaves but changed bacterial community structure, our results align more closely with the findings of Xiong et al. (2025), who observed that Bt inoculation drastically transformed the bacterial and fungal communities, specifically by balancing the ratio of Pseudomonadota to Bacillota, suppressing dominant *Staphylococcus* while promoting diverse beneficial taxa of the *Actinobacteria* and changing the relative abundance of the Ascomycota and Basidiomycota.

In this study, *Bacillus thuringiensis* and nucleopolyhedrovirus treatment triggered a marked shift from *Staphylococcus*-dominated communities (Phylum Bacillota) toward a more evenly distributed assemblage including *Pantoea* and *Pseudomonas* (Figure 4). This shift supports the competitive release hypothesis: as the biopesticide-induced environmental shift diminishes the dominance of *Staphylococcus*—a genus often associated with early-stage leaf degradation—ecological niches become available for more resilient genera within the Pseudomonadota phylum, such as *Pantoea* and *Pseudomonas*. Furthermore, the *Bacillus thuringiensis* and nucleopolyhedrovirus treatment influenced the alpha diversity indices significantly, in which the richness was significantly reduced, while diversity and evenness were enhanced (Figure 1). The reduction in richness suggests that the Bt–NPV treatment acts as a selective filter, eliminating sensitive microbial species that cannot tolerate the altered chemical environment of the treated leaf.

The same trends have been reported in tea leaves, where treatment by *Bacillus thuringiensis* changed the bacterial and fungal communities and significantly altered the Chao1 and Shannon indices (Xiong et al., 2025). Furthermore, Zhang et al. (2008) described comparable alpha diversity indices, although they indicated a significant community turnover observed by DGGE and Shannon indexes. Conversely, beta diversity analyses demonstrated a significant difference in the composition of the community in the Bt–NPV-treated and control samples, which implicated Bt–NPV treatment in directly shifting the microbial community to a different composition state (Figure 2). The observed microbiome responses to Bt–NPV treatment can be compared with the reactions of some synthetic pesticides. Zhang et al. (2023) established that when cypermethrin was used, it enhanced bacterial biomass and caused significant community turnover, whereas Bt application yielded more moderate changes.

Previous multi-omics analyses have shown strong links between microbial succession and chemical changes during cigar tobacco processing (Vorholt, 2012). Moreover, Li et al. (2024) have demonstrated that the dynamics of the microbial community are highly correlated with the chemical components of cigar tobacco fermentation; in addition, the best fermentation conditions were linked to the genera of *Pseudomonas, Pantoea*, and *Burkholderia*. Furthermore, the metagenomic and metabolomic analyses of fermentation of cigar tobacco have also revealed that the changes in the prevalent bacterial and fungal genera, including *Staphylococcus, Sphingomonas*, *Aspergillus*, and *Penicillium*, are directly associated with the changes in amino acids and aroma compounds (Yang et al., 2024). A key limitation of this study is that we did not directly measure chemical metabolites or sensory quality. Bt–NPV treatment altered the microbial community and reduced *Spodoptera litura* damage, but our current data do not indicate improved cigar tobacco quality or aroma. We did not directly study links between changes in microbes and tobacco chemicals, even though past studies have found such connections. As a result, our findings explain how leaf preservation happens in air-curing, not how quality improves. Future studies should include chemical and sensory tests to see if changes in the microbiome actually improve cigar tobacco quality.

While direct metabolomic analysis was outside the scope of this study, the sharp reduction in leaf-skeletonizing damage by *Spodoptera litura* highlights the preservation of leaf structure. In untreated controls, extensive larval feeding breaches the leaf matrix, leading to uncontrolled breakdown of essential leaf nutrients and tissue decay. In contrast, by rapidly suppressing *Spodoptera litura* populations, the synergistic Bt–NPV formulation helps maintain the leaf’s physical integrity during air-curing. Moreover, significant enrichment of beneficial bacterial and fungal groups in treated leaves, along with reduced abundance of several opportunistic taxa, suggests that Bt–NPV application may create a microbial environment favorable for leaf preservation. Still, the functional consequences of these microbiome shifts for cigar tobacco quality must be verified by direct chemical and sensory analyses.

## 5 Conclusion

This research indicated that the use of *Bacillus thuringiensis* in synergy with nucleopolyhedrovirus is an effective strategy for controlling *Spodoptera litura* both in laboratory and field environments. The *Bacillus thuringiensis* and nucleopolyhedrovirus treatment caused considerable changes in the composition of the bacterial and fungal community during the curing, while maintaining overall community diversity. The findings show the potential of Bt–NPV formulations to serve as effective and environmentally friendly *Spodoptera litura* management tools, actively modulate curing-related microbiomes, and provide an ecological framework that may support leaf preservation during air-curing. However, direct effects on cigar tobacco quality, aroma development, and chemical maturation require confirmation through integrated chemical, metabolomic, and sensory analyses. Future studies should extend the observation period beyond the initial air-curing phase to include long-term fermentation and aging. Investigating whether the early stabilization of the microbiomes by biopesticide application translates into high sensory qualities and reduced levels of tobacco-specific nitrosamines (TSNAs) over months of storage would be of high industrial relevance.

## Data Availability

The original contributions presented in the study are publicly available. This data can be found here: https://www.ncbi.nlm.nih.gov/PRJNA1484919.

## References

[ref1] Bolyen E. Rideout J. R. Dillon M. R. Bokulich N. A. Abnet C. C. Al-Ghalith G. A. . (2019). Reproducible, interactive, scalable, and extensible microbiome data science using QIIME 2. Nat. Biotechnol. 37, 852–857. doi: 10.1038/s41587-019-0209-9, 31341288 PMC7015180

[ref2] Callahan B. J. McMurdie P. J. Rosen M. J. Han A. W. Johnson A. J. A. Holmes S. P. (2016). DADA2: high-resolution sample inference from Illumina amplicon data. Nat. Methods 13, 581–583. doi: 10.1038/nmeth.3869, 27214047 PMC4927377

[ref3] Elshaghabee F. M. F. Rokana N. Gulhane R. D. Sharma C. Panwar H. (2017). *Bacillus* as potential probiotics: status, concerns, and future perspectives. Front. Microbiol. 8:1490. doi: 10.3389/fmicb.2017.01490, 28848511 PMC5554123

[ref4] Fuxa J. R. (2004). Ecology of insect nucleopolyhedroviruses. Agric. Ecosyst. Environ. 103, 27–43. doi: 10.1016/j.agee.2003.10.013

[ref5] Gupta G. P. Rani S. Birah A. Raghuraman M. (2005). Improved artificial diet for mass rearing of the tobacco caterpillar, *Spodoptera litura* (Lepidoptera: Noctuidae). Int. J. Trop. Insect Sci. 25, 1–13. doi: 10.1079/IJT200551

[ref6] Hidalgo Martinez D. Payyavula R. S. Kudithipudi C. Shen Y. Xu D. Warek U. . (2020). Genetic attenuation of alkaloids and nicotine content in tobacco (*Nicotiana tabacum*). Planta 251:92. doi: 10.1007/s00425-020-03387-1, 32242247

[ref7] Hu H. Liu Y. Huang Y. Zhang Z. Tang H. (2022). The leaf microbiome of tobacco plants across eight Chinese provinces. Microorganisms 10:450. doi: 10.3390/microorganisms10020450, 35208904 PMC8878116

[ref8] Jiang S. Q. Yu Y. N. Gao R. W. Wang H. Zhang J. Li R. . (2019). High-throughput absolute quantification sequencing reveals the effect of different fertilizer applications on the bacterial community in a tomato cultivated in coastal saline soil. Sci. Total Environ. 687, 601–609. doi: 10.1016/j.scitotenv.2019.06.105 31220714

[ref9] Li W. Yu J. Li H. Yang C. Peng Z. Zhang J. (2024). The dynamics of microbial community structure and metabolic function in different parts of cigar tobacco leaves during air-curing. Front. Microbiol. 15:1438566. doi: 10.3389/fmicb.2024.1438566, 39726961 PMC11669699

[ref10] Maharjan R. Hong S. Ahn J. Yoon Y. Jang Y. Kim J. . (2023). Temperature and host plant impacts on the development of *Spodoptera litura* (Fabricius) (Lepidoptera: Noctuidae): linear and nonlinear modeling. Insects 14:412. doi: 10.3390/insects14050412, 37233040 PMC10231086

[ref11] Maqsood S. Afzal M. Haider M. S. Khan H. A. A. Ali M. Ashfaq M. . (2019). Effectiveness of nucleopolyhedrovirus and *Bacillus thuringiensis* alone and in combination against *Spodoptera litura* (Fabricius). Pak. J. Zool. 51, 631–641. doi: 10.17582/journal.pjz/2019.51.2.631.641

[ref12] Montesinos E. (2003). Development, registration, and commercialization of microbial pesticides for plant protection. Int. Microbiol. 6, 245–252. doi: 10.1007/s10123-003-0144-x, 12955583

[ref13] Rabbee M. F. Ali M. S. Choi J. Hwang B. S. Jeong S. C. Baek K. H. (2019). *Bacillus velezensis*: a valuable member of bioactive molecules within plant microbiomes. Molecules 24:1046. doi: 10.3390/molecules24061046, 30884857 PMC6470737

[ref14] Sun Y. P. Johnson E. R. (1960). Analysis of joint action of insecticides against house flies. J. Econ. Entomol. 53, 887–892. doi: 10.1093/jee/53.5.887

[ref15] Vorholt J. A. (2012). Microbial life in the phyllosphere. Nat. Rev. Microbiol. 10, 828–840. doi: 10.1038/nrmicro2910, 23154261

[ref16] Wang X. Xue Y. Han M. Bu Y. Liu C. (2014). The ecological roles of *Bacillus thuringiensis* within phyllosphere environments. Chemosphere 108, 258–264. doi: 10.1016/j.chemosphere.2014.01.050, 24534157

[ref17] Xiong Y. Liu H. Li D. Xie W. Wang Z. Fang X. . (2025). Foliar application of *Bacillus thuringiensis* enhances tea quality and plant defense via phyllosphere microbiome modulation. Agriculture 15:1386. doi: 10.3390/agriculture15131386

[ref18] Yang Y. Pan G. Guo J. Miao C. Xu Q. Zhang Y. . (2024). The effect of flue-curing and redrying on the diversity of fungal communities in tobacco leaves. BMC Microbiol. 24:494. doi: 10.1186/s12866-024-03635-4, 39580418 PMC11585231

[ref19] Yang R. H. Su J. H. Shang J. J. Wu Y. Y. Li Y. Bao D. P. . (2018). Evaluation of the ribosomal DNA internal transcribed spacer (ITS), specifically ITS1 and ITS2, for the analysis of fungal diversity by deep sequencing. PLoS One 13:e0206428. doi: 10.1371/journal.pone.0206428, 30359454 PMC6201957

[ref20] Youssef N. Sheik C. S. Krumholz L. R. Najar F. Z. Roe B. A. (2009). Comparison of species richness estimates obtained using nearly complete fragments and simulated pyrosequencing-generated fragments in 16S rRNA gene-based environmental surveys. Appl. Environ. Microbiol. 75, 5227–5236. doi: 10.1128/AEM.00592-09, 19561178 PMC2725448

[ref21] Zhai Z. G. Hu Q. L. Chen J. R. (2020). Effects of combined application of organic fertilizer and microbial agents on tobacco soil and tobacco agronomic traits. IOP Conf. Ser. Earth Environ. Sci. 594:012023. doi: 10.1088/1755-1315/594/1/012023

[ref22] Zhang B. Bai Z. Hoefel D. Tang L. Yang Z. Zhuang G. . (2008). Assessing the impact of the biological control agent *Bacillus thuringiensis* on the indigenous microbial community within the pepper plant phyllosphere. FEMS Microbiol. Lett. 284, 102–108. doi: 10.1111/j.1574-6968.2008.01178.x 18462395

[ref23] Zhang Q. Kong G. Zhao G. Liu J. Jin H. Li Z. . (2023). Microbial and enzymatic changes during cigar tobacco air-curing and fermentation. Appl. Microbiol. Biotechnol. 107, 5789–5801. doi: 10.1007/s00253-023-12663-5, 37458766 PMC10439857

[ref24] Zong J. He X. Lin Z. Hu M. Xu A. Chen Y. . (2022). Effect of two drying methods on chemical transformations in flue-cured tobacco. Dry. Technol. 40, 188–196. doi: 10.1080/07373937.2020.1779287

